# Proposal of a Management Algorithm to Predict the Need for Expansion Duraplasty in American Spinal Injury Association Impairment Scale Grades A–C Traumatic Cervical Spinal Cord Injury Patients

**DOI:** 10.1089/neu.2022.0218

**Published:** 2022-11-30

**Authors:** Bizhan Aarabi, Chen Chixiang, J. Marc Simard, Timothy Chryssikos, Jesse A. Stokum, Charles A. Sansur, Kenneth M. Crandall, Joshua Olexa, Jeffrey Oliver, Melissa R. Meister, Gregory Cannarsa, Ashish Sharma, Cara Lomangino, Maureen Scarboro, Abdul-Kareem Ahmed, Nathan Han, Riccardo Serra, Phelan Shea, Carla Aresco, Gary T. Schwartzbauer

**Affiliations:** ^1^ Department of Neurosurgery, Division of Biostatistics and Bioinformatics, University of Maryland School of Medicine, Baltimore, Maryland, USA.; ^2^ R. Adams Cowley Shock Trauma Center, and Division of Biostatistics and Bioinformatics, University of Maryland School of Medicine, Baltimore, Maryland, USA.; ^3^ Department of Epidemiology and Public Health, Division of Biostatistics and Bioinformatics, University of Maryland School of Medicine, Baltimore, Maryland, USA.; ^4^ Department of Neurosurgery, Walter Reed National Military Medical Center, Bethesda, Maryland, USA.

**Keywords:** decompression, duraplasty, neuroprotection, spinal cord injury, surgery

## Abstract

Expansion duraplasty to reopen effaced subarachnoid space and improve spinal cord perfusion, autoregulation, and spinal pressure reactivity index (sPRX) has been advocated in patients with traumatic cervical spinal cord injury (tCSCI). We designed this study to identify candidates for expansion duraplasty, based on the absence of cerebrospinal fluid (CSF) interface around the spinal cord on magnetic resonance imaging (MRI), in the setting of otherwise adequate bony decompression. Over a 61-month period, 104 consecutive American Spinal Injury Association Impairment Scale (AIS) grades A–C patients with tCSCI had post-operative MRI to assess the adequacy of surgical decompression. Their mean age was 53.4 years, and 89% were male. Sixty-one patients had falls, 31 motor vehicle collisions, 11 sport injuries, and one an assault. The AIS grade was A in 56, B in 18, and C in 30 patients. Fifty-four patients had fracture dislocations; there was no evidence of skeletal injury in 50 patients. Mean intramedullary lesion length (IMLL) was 46.9 (standard deviation = 19.4) mm. Median time from injury to decompression was 17 h (interquartile range 15.2 h). After surgery, 94 patients had adequate decompression as judged by the presence of CSF anterior and posterior to the spinal cord, whereas 10 patients had effacement of the subarachnoid space at the injury epicenter. In two patients whose decompression was not definitive and post-operative MRI indicated inadequate decompression, expansion duraplasty was performed. Candidates for expansion duraplasty (i.e., those with inadequate decompression) were significantly younger (*p* < 0.0001), were AIS grade A (*p* < 0.0016), had either sport injuries (six patients) or motor vehicle collisions (three patients) (*p* < 0.0001), had fracture dislocation (*p* = 0.00016), and had longer IMLL (*p* = 0.0097). In regression models, patients with sport injuries and inadequate decompression were suitable candidates for expansion duraplasty (*p* = 0.03). Further, 9.6% of patients failed bony decompression alone and either did (2) or would have (8) benefited from expansion duraplasty.

## Introduction

During the past 40 years, there have been noteworthy changes in demographics and markers of injury severity in traumatic spinal cord injury (SCI).^1^ Increasingly, patients with SCI are older and have less injury severity.^2,3^ Earlier resuscitation, decompressive surgery, and multi-dimensional preventive measures may account for some of these observations; however, SCI remains one of the most difficult conditions to manage. Decompressive surgery is a credible neuroprotective measure adopted by spine surgeons.^4,5^

Under normal physiological conditions, the cervical spinal cord is immersed within cerebrospinal fluid (CSF), with a fluid interface between the spinal cord and the dura mater, outside of which reside the skeletal elements and the disco-ligamentous complex (DLC). As such, intraparenchymal pressure is equal to the CSF pressure of the subarachnoid space (SAS).^6,7^ Any increase in volume of the spinal cord because of post-traumatic hemorrhage or cytotoxic/vasogenic edema gives rise to a proportional displacement of CSF.

The swollen spinal cord can expand to occupy the entire cross-sectional area of the thecal sac, resulting in loss of the SAS at a given injury level.^8^ Because the dura mater and skeletal elements have minimal viscoelasticity, further swelling will be followed by an increase in intraparenchymal pressure and a compromise of spinal cord perfusion pressure (SPP), autoregulation, and spinal pressure reactivity index (sPRx). Variations in intraspinal pressure (ISP) and any need for expansion duraplasty must be verified by ISP monitoring.^9^

In primary blunt traumatic cervical spinal cord injury (tCSCI), there may be an abrupt, precipitous failure of skeletal elements resulting in translocation of bone fragments and DLC into the spinal canal, impacting the spinal cord and the dura mater, with immediate disappearance of the intervening CSF interface. On magnetic resonance imaging (MRI), deformation of the spinal cord is interpreted as *extrinsic pressure*. These changes can lead to axonal injury, disruption of the vasculature, compromised perfusion, and ischemia. Extrinsic pressure or a drop in perfusion pressure should be remedied accordingly.^9–15^

Swelling of the spinal cord induces *intrinsic pressure* against an unyielding dura, further effacement of the CSF interface, a precipitous surge in ISP, and further decline in SPP.^16^ Pre-clinical and limited clinical studies have shown proportional improvements in neurological function and in evoked potentials when extrinsic pressure on the spinal cord was remedied in a timely manner, suggesting that prompt surgical decompression in humans may be neuroprotective.^5,17–22^ Surgical decompression of extrinsic and intrinsic forces impacting the spinal cord requires further study.

Much of the literature centered on extrinsic decompression of the spinal cord after blunt tCSCI was based on anatomical realignment of the cervical spine by traction reduction and/or removal of the compressive boney elements followed by internal fixation.^23^ In general, no MRI was performed to document that the techniques utilized were effective in achieving total extrinsic and intrinsic spinal cord decompression.^5,24^ Recent evidence indicates that, in patients with motor complete cervical SCI, when MRI was utilized to assess the spinal cord post-operatively and “complete decompression” was defined as the presence of CSF on either ventral, dorsal, or ventral/dorsal surface of the spinal cord, discectomy or corpectomy without laminectomy resulted in decompression of the spinal cord in only 46.8% to 58.6% of cases.^8^

The addition of laminectomy to complement anterior surgery improved the rate of decompression up to 73.1%. In addition, as the number of laminectomy segments increased, so too did the rate of complete decompression, re-establishing a patent SAS/CSF interface on the ventral, dorsal, or ventral/dorsal surface of the swollen spinal cord. Complete decompression was associated with improved American Spinal Injury Association (ASIA) Impairment Scale (AIS) grade conversion.^8,15^

Since 2015, real-time ISP monitoring has indicated that in a significant number of patients with severe SCI (AIS grades A–C), bony decompression is insufficient to achieve complete spinal cord decompression, and therefore expansion duraplasty is required to re-establish a patent SAS and to improve SPP, sPRx, and autoregulation. In addition, improved spinal cord perfusion improves the ASIA motor score (AMS).^6,25^

We hypothesized that in tCSCI patients with AIS grades A–C, adequate laminectomy offers complete *extrinsic* and *intrinsic* spinal cord decompression in the majority of patients, and that expansion duraplasty is required in only a minority of subjects.

## Methods

### Design

The study is a retrospective analysis of prospectively collected data.

### Primary objective

The objective of the study was to determine indications for expansion duraplasty in addition to adequate laminectomy in patients with AIS grades A-C tCSCI.

### Cohort

From 1 January 2017 to 1 February 2022, 432 patients with tCSCI were admitted to this Level I Trauma Center, of whom 164 patients were screened for inclusion in this study. Patients presenting with AIS grades A, B, and C were included. The extent of the posterior cervical decompression was based on the intramedullary lesion length (IMLL)—one level of laminectomy was prescribed for each 15 mm (the height of a single vertebral body) of IMLL. Laminectomy was performed in addition to anterior cervical discectomy and fusion (ACDF) or anterior cervical corpectomy and fusion. Decompression was judged adequate if, on post-operative MRI or computed tomography (CT) myelogram, CSF was visualized ventral or dorsal or both ventral and dorsal to the spinal cord.

Subjects were excluded if they had only anterior surgery without laminectomy (21 patients) or if they had inadequate laminectomy—i.e., continued compression of the spinal cord at the rostral and/or caudal skeletal segments (10 patients). In addition, patients were excluded if tCSCI was because of penetrating injury (13 patients) and if the level of injury was restricted to upper cervical spine segments (C1/C2, 10 patients). No contrast or MRI studies, spinal epidural hematoma, SCI without radiological abnormality, and non-operative management were other reasons for exclusion (six patients). Post-operative CT myelogram (in three patients) and MRI confirmed adequate laminectomy in 104 patients. The research started after approval by the University of Maryland HRPO, IRB office (HP-00100211).

### Trauma Resuscitation Unit (TRU)

Once the patients were medically stable after initial resuscitation,^26^ a neurosurgery team (senior resident, nurse practitioners with or without attending neurosurgeon) performs a complete neurological assessment. Admission AMS, AIS grades, neurological level of injury (NLI) are determined according to the International Standards for Neurological Classification of Spinal Cord Injury (ISNCSCI).^27^

### CT, MRI, and real-time intraoperative ultrasonography (IOUS)

For patients in this study, multi-planar cervical spine CT scan was performed within a median of 2 h of trauma, and multi-planar multi-sequence T2-weighted and short T1 inversion recovery (STIR) sequences were acquired within a median of 7 h after trauma. We defined the injury type morphology of the cervical spine fracture dislocations according to the Vaccaro and associates^28^ AO Spine Classification system.

Intramedullary lesion length was measured on sagittal T2 and/or STIR MRI.^15^ Midsagittal CT was compared with midsagittal MRI sequences to determine the adequacy of laminectomy, surgical technique, and the extent of spinal cord decompression after surgery.^29^ Complete decompression was defined as presence of a cerebrospinal fluid interface ventral, dorsal, or ventral/dorsal surface of the spinal cord and dura continuously from the foramen magnum to the first thoracic vertebra on midsagittal cuts.

Two spine fellowship-trained neurosurgeons (KC and CS) and two neurosurgeons (GS and BA) validated the extent of laminectomy and spinal cord decompression after surgery. Any discrepancies were adjudicated by a consensus conference of the four neurosurgeons.^15,30^ The Principal Investigator (PI) measured the IMLL on pre-operative MRI images.^15^ From July 2021, we incorporated in situ real-time IOUS in our armamentarium to verify decompression after completion of surgical intervention in patients who had laminectomy. Thus, we could compare decompression via IOUS and post-operative MRI.

### Blood pressure augmentation and steroid protocol

Patients’ mean arterial blood pressure (MAP) was maintained between 85 to 90 mm Hg for seven days after trauma when medically feasible.^31,32^ Steroid protocol has not been in use at this medical center since 2010.^29^

## Traction reduction

If the admission CT scan indicated a need for traction reduction of fracture dislocation, this procedure was performed in the TRU with the patient placed on a Stryker frame (Kalamazoo, MI) and using a standby image intensifier. Traction reduction was performed after resuscitation in 28 patients. From this cohort, four patients had compression teardrop fractures, seven patients had unilateral locked facets, and 17 patients had bilateral locked facets. Traction was not indicated in 76 patients. Morphology was AO Spine injury type A0, i.e., no visible fracture dislocation, in 52 patients; AO Spine injury type B, i.e., flexion or extension distraction without translation, in 11 patients; and compression teardrop or burst fracture in 11 patients. Open reduction was performed in two unilateral locked facet patients.

### Surgical intervention and expansion duraplasty

Surgical decompression, in addition to deformity correction, was in one or multiple stages. Pre-operative planning for de facto decompression of the spinal cord was based heavily on pre-operative IMLL in addition to injury morphology. Based on our previous (2001–2016) experience,^8^ we prescribed laminectomies involving multiple motion segments, corresponding in length to the IMLL—i.e., one level of laminectomy for each 15 mm (the height of one vertebral body) of IMLL regardless of one or multiple levels of ACDF or anterior cervical corpectomy and fusion (ACCF). If a patient had ACDF or ACCF (e.g., for locked facets or compression fracture), laminectomy was performed as a second stage of decompression based on IMLL. The first stage of surgical intervention started a median of 17 h after trauma (interquartile 15.2 h).

### Critical care management

After admission to the critical care unit, enoxaparin (Lovenox®, Sanofi, USA) 30 mg twice daily was administered starting within 24–48 h of trauma for deep vein thrombosis (DVT) prophylaxis. In addition, screening for DVT by duplex ultrasound was performed when there was clinical indication. Early tracheostomy and gastroenterostomy for nutrition were performed when indicated.

### Follow-up of patients

This research was an acute care investigation.

### Statistical analysis

#### Statistical methods

Marginal associations between decompression, accident, gender, age, AIS grade, AMS, morphology, and IMLL were analyzed using the Fisher exact test (if categorical) and the Student *t* test (if numerical). Conditional associations were investigated using logistic regression by the Jeffreys prior.^33^ This penalized logistic regression (also named the Firth method) outperforms the conventional logistic regression and leads to less biased estimates under the data with small samples and several unbalanced and highly predictive risk factors.^34^ The statistical analysis was performed using Software :R version 4.1.0

## Results

### Mechanism of injury

Overall, 10 patients were not completely decompressed on post-operative MRI with no visible CSF on ventral, dorsal, or ventral/dorsal surface of the cord post-operatively. Uni- and multi-variate analysis indicated that patients with recreational sport injuries were more prone to excessive cord swelling not easily relieved by decompressive surgery. Six of 11 sport injuries (54.5%) were not completely decompressed in post-operative MRI images. Five of six patients had shallow-dive injuries, and one was injured in a friendly bout of wrestling. The mean IMLL in this group of patients was 72.1 (18.3) mm.

**FIG. 1.**
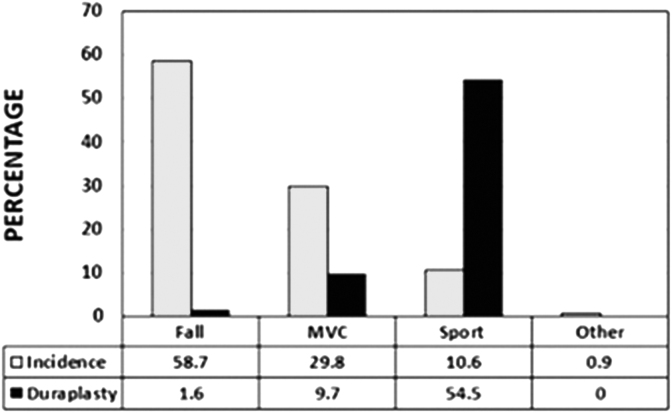
Graph indicating the relationship between mechanism of injury and the percentage of patients who could benefit from duraplasty for complete spinal cord decompression. MVC, motor vehicle collision.

Three of 31 patients injured in a motor vehicle collision (MVC) failed to have complete decompression (i.e., presence of CSF on ventral, dorsal, or ventral/dorsal surface of the cord with laminectomy in conjunction with discectomy+/-corpectomy. The mean IMLL in this group was 45 mm. Similarly, among 61 patients with ground-level falls, one patient (1.6%) did not have complete decompression on post-operative MRI with decompressive techniques. The IMLL in these patients measured 45.2 mm (Fig. 1).

### AIS grade

Fifty-five patients in this investigation were in the AIS grade A group with an AMS of 9.3 (standard deviation [SD] = 9.5), 18 were in the AIS grade B group with an AMS of 10.8 (SD = 10.4), and 31 were in the AIS grade C group with an AMS of 23.7 (SD = 17.4). The IMLL in AIS grade A patients was 55.1 (SD = 18.9); in AIS grade B patients, 38.5 (SD = 12.5); and in AIS grade C patients, 37.0 (SD = 17.5). All 10 patients suitable for expansion duraplasty had complete quadriplegia (AIS grade A), with an IMLL of 56.4 (SD = 20.3) mm.

### CT and MRI morphology

When injury morphology was classified according to Vaccaro and associates^28^ AO Spine Classification, 50 patients did not have any visible fracture dislocations (AO Spine injury type A0), 46 had either translation rotation (AO Spine injury type C), or compressive flexion injuries (AO Spine injury type A4). Eight patients had either extension or flexion distraction injuries with no translation (AO Spine injury type B2 or B3). All 10 patients with swelling not relieved by decompressive laminectomy with or without ACDF or ACCF were in groups C or A4 injuries.

### IMLL on MRI

The IMLL, as detected by T2-weighted or STIR MRI, is strongly related to tCSCI severity and parenchymal swelling. In motor complete tCSCI (AIS grades A and B), the rate of expansion of IMLL after trauma is close to 900 μm/h.^14^ The IMLL may signify the extent of intrinsic pressure on the spinal cord. Younger patients, patients injured while participating in recreational sports, and those sustaining complete quadriplegia because of tear-drop fractures or unilateral locked facets were prone to not having a complete decompression on post-operative imaging despite undergoing a decompressive laminectomy.

### Extent of intrinsic decompression

On post-operative imaging, 94 of 104 patients were found to have achieved a “complete decompression” as defined previously, with CSF interface visible along the ventral, dorsal, or ventral-dorsal surface of the spinal cord. Median time interval from injury to post-operative MRI in 104 patients was 37 h (range 12–661 h, interquartile range [IQR] 27.3 h). Median time to post-operative MRI in decompressed patients was 37 h and in those not decompressed, 35 h).

**FIG. 2.**
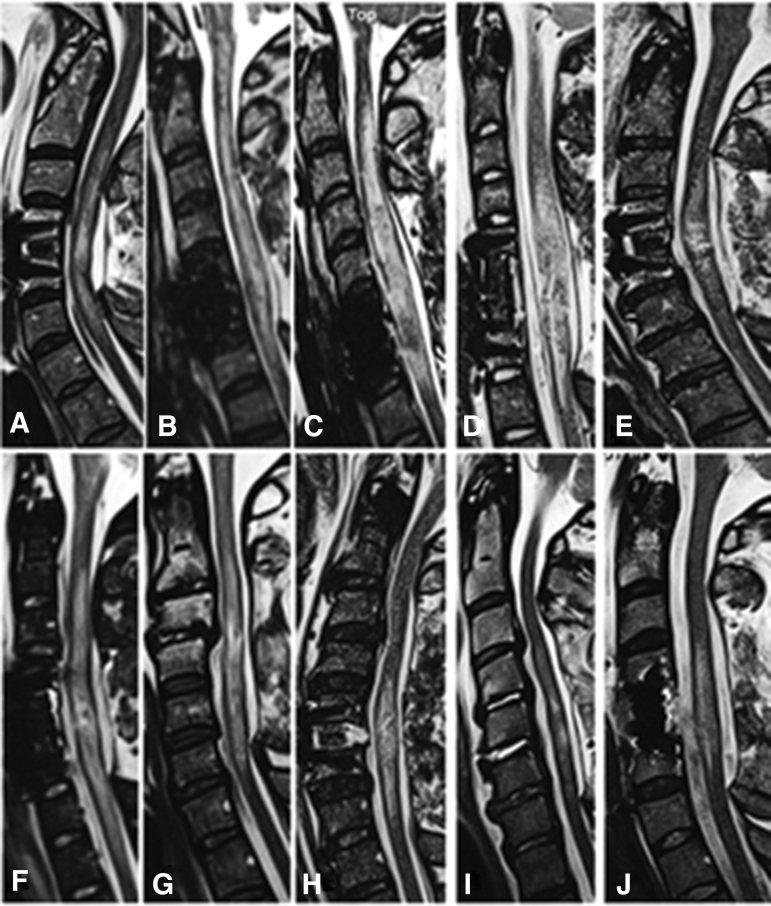
Post-operative midsagittal magnetic resonance imaging (MRI) views from 10 randomly selected patients with adequate decompression plates **(A–J)**. Adequate decompression was defined as presence of cerebrospinal fluid (CSF) interface in front, behind, or in front/behind of the spinal cord. In panels A, C, and F, the CSF interface is partially missing behind the spinal cord, which might be because of the effect of gravity while the patient is in the supine position in the MRI gantry.

Surgical intervention included 14 patients with ACCF+laminectomy+posterior spinal fusion (PSF); 39 with ACDF+laminectomy+PSF; and 51 with stand-alone laminectomy+PSF (Fig. 2). Pre-operative IMLL was 54.4 mm in the corpectomy group, 48.3 in the ACDF group, and 43.7 in the laminectomy group. Among the three groups, the number of laminectomy levels was 3.6 segments in the corpectomy group, 3.2 segments in the discectomy group, and 3.4 segments in the laminectomy group.

**FIG. 3.**
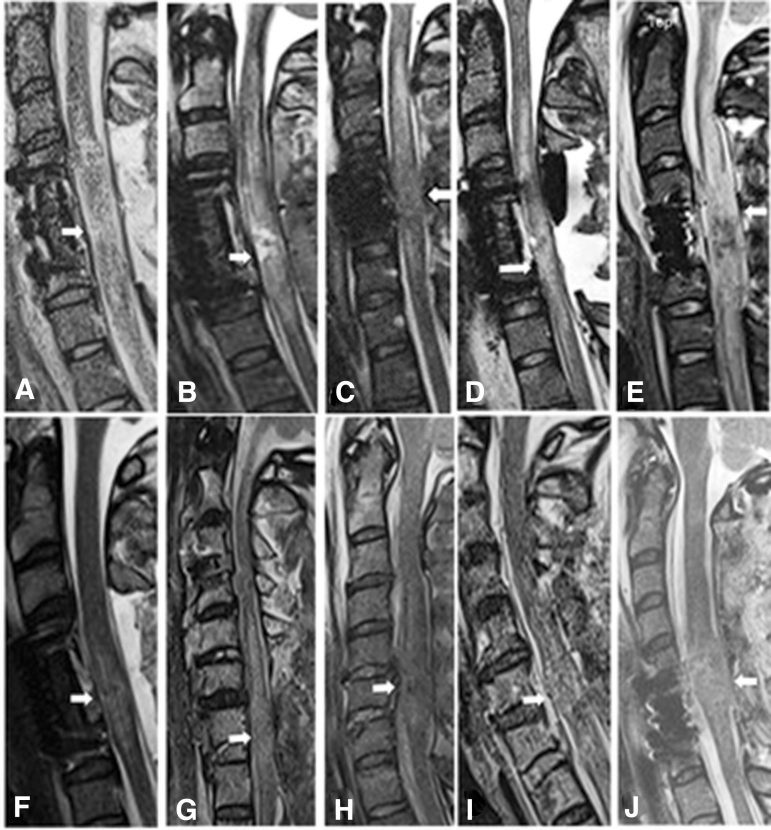
Post-operative midsagittal magnetic resonance imaging views from 10 patients with inadequate decompression despite anterior cervical corpectomy and fusion (ACCF) and laminectomy (plates A, B, D, E, F, J), anterior cervical discectomy and fusion, and laminectomy (plate C), and standalone laminectomy with posterior spinal fusion (plates G, H, I). The cerebrospinal fluid (CSF) is not visible either dorsal or ventral to the spinal cord indicating excessive swelling. Although intraspinal pressure was not unknown, in patients B and C, expansion duraplasty was performed to generate a CSF interface between the spinal cord and dura.

Real-time IOUS in 17 consecutive patients who had surgery from July 2021 through January 2022 indicated adequate decompression in 15 patients. In this group of patients, decompression was verified by post-operative MRI. In two patients, real-time IOUS revealed that there was barely a rim of visible CSF around the spinal cord, but on post-operative MRI, it was thought that there was not a complete decompression. These two patients were re-operated on to have expansion duraplasty. These two patients were among the 10 patients with inadequate decompression. The IMLL in these patients was 61.8 mm, and mean number of laminectomies with PSF 4.2 levels (Fig. 3B, C). 

### Statistical interpretation

For univariate analysis, samples were separated according to adequate or inadequate decompression. We evaluated the distribution of risk factors between these two populations. Variables such as accidents (*p* < 0.0001), age (*p* < 0.0001), AIS grade (*p* = 0.0016), morphology (*p* = 0.00016), and IMLL (*p* = 0.0097) were detected to be significant (Table 1). These variables were subsequently probed using multi-variate analysis based on the logistic regression by Jeffreys prior (Table 2). It became clear that sport injuries (odds ratio [OR] = 0.09, *p* = 0.03) were significant, and AIS grade A was marginally significant (OR = 0.08, *p* = 0.07).

**Table 1.** Baseline Characteristics of the Present Cohort

	Number of patients with inadequate decompression (%)	Number of patients with adequate decompression (%)	Total number (%)	*p*
Accident Fall MVC Sport Other Total	1 (10) 3 (30) 6 (60) 0 (0) 10 (9.6)	60 (63.8) 28 (29.7) 5 (5.3) 1 (1.1) 94 (90.4)	61 (58.7) 31 (29.8) 11 (10.6) 1 (0.9) 104 (100)	*<* 0.0001
Gender Male Female Total	9 (90) 1 (10) 10 (9.6)	80 (85.1) 14 (14.9) 94 (90.4)	89 (85.6) 15 (14.4) 104 (100)	*1.00*
Age (years): Mean (SD)	33.1 (15.6)	55.6 (15.6)	53.4 (16.9)	*<* 0.0001
AIS grade (%) AIS A AIS B AIS C	10 (100) 0 (0) 0 (0)	46 (48.9) 18 (19.2) 30 (31.9)	56 (53.8) 18 (17.3) 30 (28.9)	0.0016
ASIA motor score: Mean (SD)	10.7 (13.8)	14.2 (13.1)	13.8 (13.1)	0.43
Morphology (%) A0 C B Total	0 (0) 10 (100) 0 (0) 10 (9.6)	50 (53.1) 36 (38.2) 8 (8.5) 94 (90.4)	50 (48.1) 46 (44.2) 8 (7.7) 104 (100)	0.00016
IMLL (mm): Mean (SD)	61.8 (20.3)	45.5 (18.7)	46.9 (19.5)	0.0097

MVC, motor vehicle collision; SD, standard deviation; AIS, American Spinal Injury Association (ASIA) Impairment Scale; IMLL, intramedullary lesion length.

**Table 2.** Multiple Regression (Penalization of the Logistic Regression by the Jeffreys Prior

	Coef	SE	OR	LL-95% CI	UL-95% CI	*p*
Accident	-2.44	1.13	0.09	0.01	0.80	0.03
AIS grade	-2.48	1.39	0.08	0.01	1.28	0.07
Morphology	-1.94	1.32	0.14	0.01	1.89	0.14
Age	-0.00	0.03	1.00	0.95	1.05	0.99
IMLL	-0.00	0.02	1.00	0.95	1.04	0.93
Gender	0.57	1.04	1.77	0.23	13.55	0.58

SE, standard error; OR, odds ratio; LL, lower limit; UL, upper limit; CI, confidence interval; AIS, American Spinal Injury Association Impairment Scale; IMLL, intramedullary lesion length.

## Discussion

Surgical decompression as a neuroprotective measure after tSCI is gaining momentum among spine surgeons.^5,22,29,35–38^ By removing all extrinsic and intrinsic pressure on the spinal cord in conjunction with anatomic realignment and fusion, ISP may equalize with CSF pressure, augment SPP, and support autoregulation. The technique of adequate decompression and the role of expansion duraplasty remains to be defined, however.^16, 25, 37-41^

Evidence from our investigation indicated that adequate laminectomy (irrespective of anterior technique) based on IMLL obviated the need for expansion duraplasty in nearly 90% of surgical patients. We divided the pre-operative IMLL into 15 mm segments (the height of a single cervical spine vertebra) and took the resulting number as the number of levels of laminectomy needed to attain adequate decompression. Subjects who needed duraplasty were significantly younger and sustained quadriplageia after shallow dives or MVCs with obvious fracture-dislocations. Notably, IMLL in these patients was significantly longer than the IMLL recorded in older patients manifesting incomplete injuries from ground-level falls and without clear-cut skeletal injuries.

**FIG. 4.**
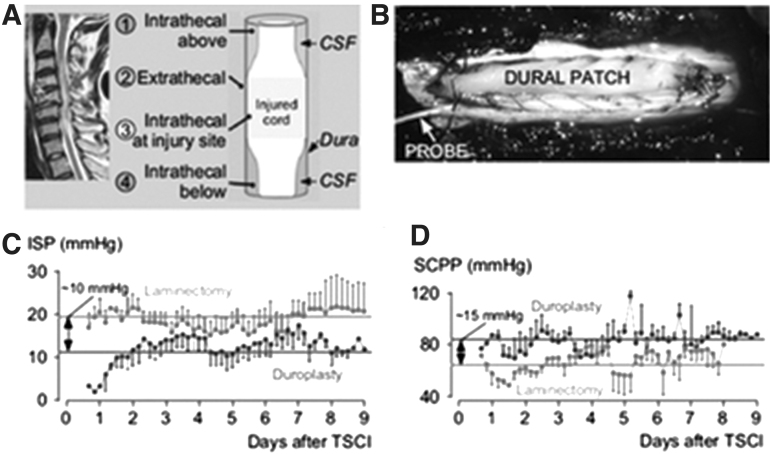
Proposed expansion duraplasty aimed at reducing intraspinal pressure (ISP) and improving spinal cord perfusion pressure (SCPP) and spinal pressure reactivity index and autoregulation Plates **A–D** (after Saadoun with permission Neurotherapeutics 17:511-521, 2020^38^). CSF, cerebrospinal fluid; tSCI, traumatic spinal cord injury.

Real-time multi-modality pressure and neurochemical monitoring studies at St. George's Hospital in the United Kingdom (Fig. 4) have indicated that under normal physiological conditions, when a swollen spinal cord has not displaced the entire CSF interface, ISP is equal to CSF presssure. If the swollen spinal cord becomes intrinsically compressed within the dura mater, however, it has been shown that expansion duraplasty can lower the ISP and improve perfusion pressure, sPRx, and autoregulation. Detailed studies from this institution have indicated that laminectomy without duraplasty will not normalize ISP in a subset of AIS grade A–C patients.^36,41–48^

We carefully reviewed post-operative midsagittal MRI studies of the 10 patients presented in this study who lacked a patent CSF interface despite adequate bony decompression. For these patients, there was severe swelling of the spinal cord probably causing intrinsic pressure against the dura. Still, some extrinsic pressure by serosanguinous fluid or air (Fig. 3, plates A, D, and F) or swollen or tightly sutured muscle (Fig. 3, plates B and H) could not be ruled out.

Our present algorithm for elimination of extrinsic and intrinsic pressures on the swollen spinal cord is based on our pre-operative planning after careful consideration of injury morphology, IMLL, and traction reduction when applicable. As such, in AIS grades A-C patients in addition to anatomical alignment and internal fixation, as recommended by Saadoun and colleagues,^16 25,36,39^ three to five levels of laminectomy are required for total decompression. Under such conditions, lumbar CSF pressure might be taken as ISP, a parameter by which we can adjust our SPP and sPRx.^7,9,36,47^

**FIG. 5.**
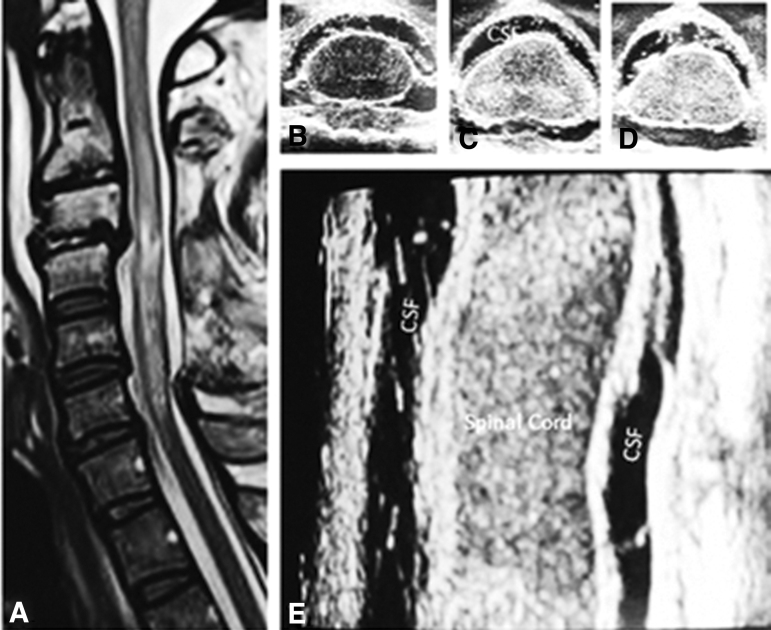
Midsagittal post-operative magnetic resonance imaging view of a 58-year-old man with motor vehicle collision and an American Spinal Injury Association (ASIA) Impairment Scale (AIS) grade C (ASIA motor score 10) spinal cord injury indicating hematomyelia, cord swelling (intramedullary lesion length 62.5 mm), and complete decompression of the spinal cord as evidenced from the cerebrospinal fluid (CSF) interface in front and in the back of the spinal cord (plate A). The composite plates on the right side are real-time intraoperative ultrasonography with presence of CSF around (plates B, C, and D), in front, and the back of the spinal cord (plate E).

With real-time imaging available at the time of surgery, we are beginning to take advantage of IOUS to determine the need for expansion duraplasty. The patient in Figure 5 was a 58-year-old man who had a MVC and was admitted conscious with an AIS grade of C and an AMS of 10. The CT scan and MRI were compatible with spinal stenosis and an IMLL of 62.5 mm. At 21 h after trauma, he had a three-level laminectomy with complete decompression noted on IOUS and post-operative MRI.

**FIG. 6.**
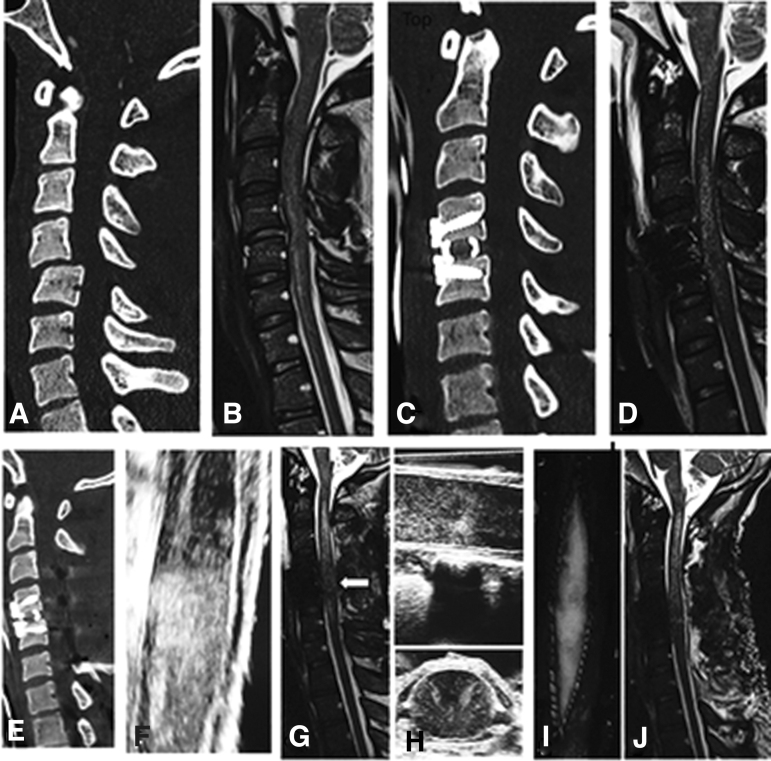
Midsagittal computed tomography and magnetic resonance imaging (MRI) views (plates A and B) from a 20-year-old male patient with C4/5 unilateral locked facets and quadriplegia during a friendly game of wrestling. Plate A indicates translation rotation injury between C4 and C5 (AOSpine injury type C^28^) vertebral bodies 6 h after trauma. Nine hours after traction reduction, the MRI indicated lack of cerebrospinal fluid (CSF) interface in front and in the back of the spinal cord from C2–C6 and an intramedullary lesion length of 73.1 mm. (plate B). After anterior cervical discectomy and fusion, MRI (plate D) indicated continued absence of CSF in front and in the back of the spinal cord. The patient had a second stage surgery that was laminectomy. Real-time intraoperative ultrasonography after laminectomy revealed spinal cord swelling with a thin interface in front of the spinal cord; however, the surgeon was not convinced expansive duraplasty was necessary (plates E and F). Post-operative MRI (plate G) revealed absence of CSF around the spinal cord at C4/5 skeletal segments. Although intraspinal pressure monitoring was not available at the time, the patient was re-explored for expansion duraplasty (plates H, I, and J), which re-established CSF interface around the spinal cord.

The patient in Figure 6 was a 20-year-old male who sustained complete quadriplegia after an episode of wrestling. Five hours after the episode, he was conscious, his AMS was 8, and AIS grade was A. After traction reduction of his unilateral locked facet and internal fixation, on the second day of admission he underwent four-level laminetomy. Real time IOUS indicated CSF interface between dura and cord (pictures not available); duraplasty was not performed. Post-operative MRI indicated persistent intrinsic swelling. The patient was re-explored and had expansion duraplasty. The IOUS in this stage of surgery indicated effaced spinal cord.

Real-time IOUS may obviate the need for routine post-operative MRI to prove adequate decompression. Importantly, real-time IOUS can help imaging hematomyelia, epidural, or subdural hematomas.^49^

### Limitations

There are multiple limitations to this study. Besides being a retrospective, uncontrolled, and a single center investigation, because we did not have ISP in these patients, we are unable to conclude decisively that there was increased pressure within the cord when the spinal cord is observed to be touching the dura on the post-operative MRI. In addition, ISP monitoring as a support for expansion duraplasty has its own limitations of feasibility, complications, and inadequate validated outcome studies. The randomized controlled trial study of DISCUS^48^ may help resolve some of these uncertainties.

## Conclusion

Relief of extrinsic and/or intrinsic pressure after tCSCI is a neuroprotective strategy achieved through surgical decompression, including bony decompression and, where necessary, expansion duraplasty. Based on our extensive studies, in AIS grades A–C patients, a complete decompression is most likely achieved with multi-level laminectomy taking into consideration the IMLL subjacent to the corresponding cervical skeletal segments. Dividing the IMLL into 15 mm segments will result in the number of needed levels of laminectomy for the intended complete decompression—i.e., re-established CSF interface anteriorly, posteriorly, or in anterior/posterior aspects of the swollen spinal cord. In a small subset of patients, however, effacement of the swollen spinal cord against the dura may not be relieved with the projected laminectomy unless an expansion duraplasty is performed.
